# Selective Extraction of Bioactive Phenylethanoids from *Digitalis obscura*

**DOI:** 10.3390/plants10050959

**Published:** 2021-05-12

**Authors:** José Francisco Quílez del Moral, Álvaro Pérez, María José Segura Navarro, Alberto Galisteo, Azucena Gonzalez-Coloma, María Fe Andrés, Alejandro F. Barrero

**Affiliations:** 1 Department of Organic Chemistry, Institute of Biotechnology, University of Granada, 18071 Granada, Spain; alvaroapr@ugr.es (Á.P.); mariajoseseguranavarro@gmail.com (M.J.S.N.); albertogapre@ugr.es (A.G.); 2 Institute of Agricultural Sciences, CSIC, 28006 Madrid, Spain; azu@ica.csic.es (A.G.-C.); mafay@ica.csic.es (M.F.A.)

**Keywords:** selective extraction, natural products, pesticides, antifeedant, nematicidal

## Abstract

Cardenolide-free extracts from *Digitalis obscura* showed significant antifeedant effects against the aphid *Myzus persicae* and this activity correlated with their phenylethanoid content. The content in phenylethanoids of *Digitalis obscura* has been studied. Maceration of the aerial parts of *D. obscura* was used for the selective extraction of the natural compound rengyolone (**1**) and the aglycone of cornoside (compound **3**). Pure rengyolone (**1**) can be obtained from *D. obscura* in approximately 90% purity from fresh plant from the CHCl_3_ soluble fraction of the ethanolic extract (0.8% yield). The ethanol extraction of freshly collected *D. obscura* showed the presence of compound **3** as the only phenylethanoid. Compound **3** was proven to easily evolve to rengyolone. Due to this instability, and although its presence in plants has been previously reported, the spectroscopical data of **3** are reported herein for the first time. Selective mono-acetylation of compound of **3** led to the active natural compound hallerone (**5**). The aphid antifeedant (against *Myzus persicae*) and nematicidal (against root-knot nematode *Meloidogyne javanica*) activities of these compounds have been evaluated. Here we report for the first time on the aphid antifeedant effects of **1**, **3**, and **5**. Additionally, the nematicidal activity of hallerone (**5**) is described here for the first time.

## 1. Introduction

Food safety and environmental concerns related to the use of pesticides have resulted in more restricted regulatory frameworks worldwide, reducing the number of commercial products available for crop protection. Therefore, new, safer, and effective protection agents are needed. Natural products have been known for decades as crop protection agents [1,2,3,4]. Many active ingredients of botanical pesticides, such as azadirachtin or essential oils, come from medicinal plants [5]. 

During the last decade, our research groups have contributed to the discovery of new natural or natural-derived biopesticides from medicinal plants [6], and some of these compounds have been patented based on their activity [7].

Among traditional medicinal plants, the genus *Digitalis* contains species used for the treatment of congestive heart failure [8]. Cardenolides are the pharmacologically active components of *Digitalis* [9] and well-known plant defenses sequestered by specialist insects that deter feeding in nonadapted insects [10] and have nematicidal effects [11] depending on their molecular structure. In addition to toxic cardenolides, Digitalideae contain phenylethanoids, such as rengyolone (**1**) and cornoside (**2**), which are considered chemotaxonomic markers [12]. Rengyolone (or halleridone) (**1**) has a wide array of pharmacological activities, including cytotoxicic [13,14,15], anti-inflammatory [16], antiplasmodial [17], and antioxidant [18] while cornoside (**2**) has reported cytotoxic effects [19]. However, little is known on the plant defensive properties of these compounds.

*Digitalis obscura* is an endemic medicinal plant growing in the Iberian Peninsula and Northern Morocco. In the province of Granada (Andalusia, Spain), this species has been used in ethnoveterinary practices, specifically for traumatic lesions and poisoning [20]. *D. obscura* contains toxic cardenolides [21,22], and its cultivation in vitro has been extensively studied [23]. However, little is known about its content in additional metabolites involved in plant defense with lower toxicity to vertebrates.

In this study, we have explored the presence of phenylethanoids in *D. obscura* to search for new plant defense-based biopesticide models. Cardenolide-free *D. obscura* extracts have been prepared, phenylethanoids have been selectively extracted and the plant protection properties (insect antifeedant effects against *Spodoptera littoralis*, *Myzus persicae* and *Rhoplaosiphum padi*, and nematicidal action against *Meloidogyne javanica*) of these extracts and products tested. 

## 2. Results and Discussion

### 2.1. Digitalis Obscura Cardenolide-Free Extracts and Their Biological Effects

The selective extraction of natural products from their sources represents a determining step in terms of efficiency and viability [24,25]. All this makes the search of protocols for the selective extraction of natural products a demanding task. Here we have developed a selective extraction method to obtain cardenolide-free *D. obscura* extracts and the natural compound regyolone (**1**).

Aerial parts of *Digitalis obscura* collected in July 2020 were used to obtain plant extracts by maceration using solvents of different polarity such as *tert*-butylmethyl ether (MTBE), ethyl acetate (EtOAc) or ethanol (EtOH). Additionally, extracts were also obtained from the powdered aerial parts with 70% EtOH using a Soxhlet extractor (reflux). The extracts content was qualitatively analyzed by NMR (Table 1). 

The conventional EtOH:H_2_O extraction of the powdered aerial parts of the plant yielded glycosylated phenylethanoids and free sugars as major compounds, along with minor proportions of cardenolides (entry 1, Table 1). Similar results were found when the fresh aerial parts were extracted again with refluxing EtOH (100%), although a moderate increase in the ratio of phenylethanoids was noticed (entry 2, Table 1).

With the aim of achieving a selective extraction of non-glycosylated phenylethanoids, extractions of the aerial parts using less polar solvents and/or lower temperatures were tested (entries 3–6, Table 1). In entries 3–4, although the presence of non-glycosylated phenylethanoids was observed, fats were the major compounds in the extract. The use of EtOH at 40 °C (entry 5, Table 1) led to the selective extraction of phenylethanoids.

The crude extracts containing phenylethanoids (entry 3-MTBE and entry 6-ethanolic, Table 1) were tested against insect pests (*Spodoptera littoralis*, *Myzus persicae* and *Rhopalosiphum padi*) and phytoparasitic nematodes (*Meloidogyne javanica*) of great economic importance. Thus, the moth *S. littoralis*, also known as Egyptian cotton leafworm, was reported to be a pest of this plant and of a wide variety of vegetable crops [26]. The aphid *M. persicae* is known to infect a large number of species from 40 plant families, with special prevalence in *Brassica* crops [27]. *R. padi* is also an aphid and is considered one of the most important cereal pests [28]. Root-knot nematodes (*Meloidogyne* sp.) are plant parasites of major agricultural importance [29]. Only *M. persicae* was significantly affected by these extracts (3 doses, 88–42 and 74–45%SI) with EC_50_ values of 40.1 (29.5–54.4, 95% CL) and 27.9 (10.58–49.5, 95% CL) µg/cm^2^, respectively. Extract 3 (MTBE) contained more phenylethanoids than extract 5 (ethanolic) (Table 1), suggesting a correlation between the phenylethanoid content of the extract and the aphid antifeedant effect.

### 2.2. Selective Extraction of Rengyolone (***1***)

**Figure 1:**
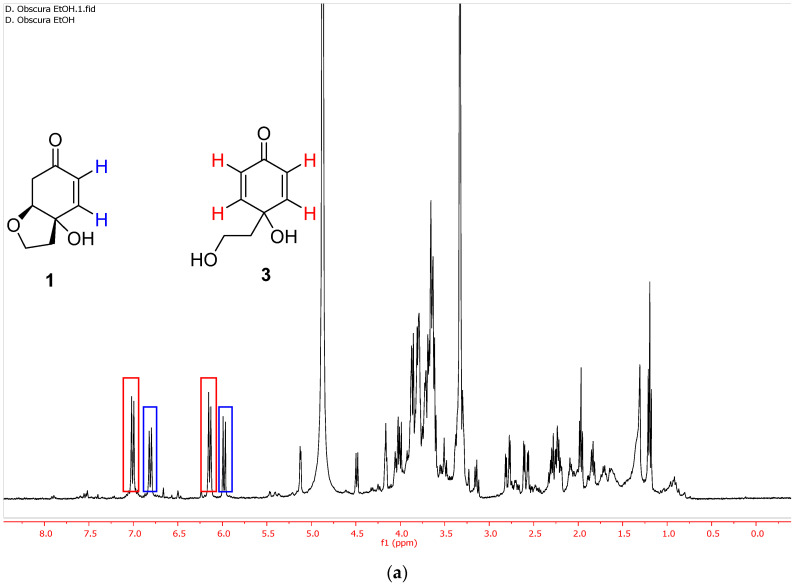

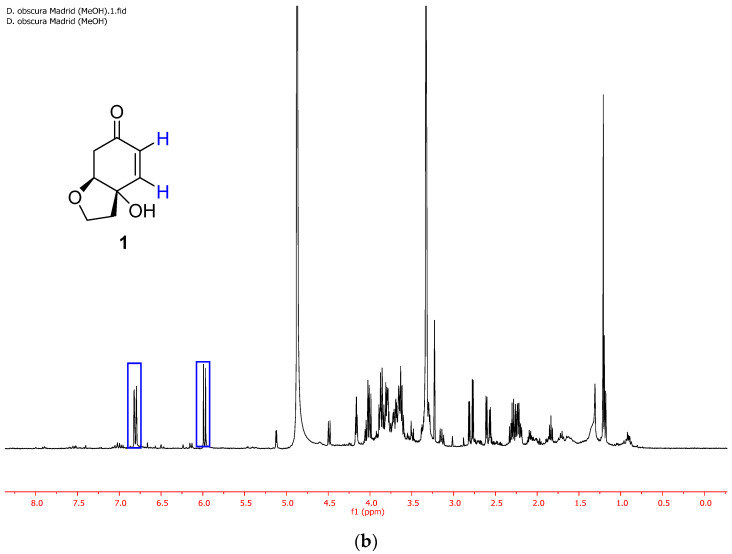
(**a**) ^1^H NMR spectrum of the ethanolic extract of *D. obscura* just after the extraction (entry 5, Table 1). (**b**) ^1^H NMR spectrum of the same ethanolic extract of *D. obscura* a few weeks after the extraction.

The extraction with EtOH at 40 °C (entry 5, Table 1) led to the selective extraction of phenylethanoids, which consisted in a mixture rengyolone (**1**) and compound **3**, the aglycone of the known phenylethanoid cornoside (**2**) (Figure 1a). Regarding these two compounds, it was noticed that upon standing for a few weeks in the freezer, diol **3** evolved completely to rengyolone (**1**) (Figure 1b). 

**Figure 2:**
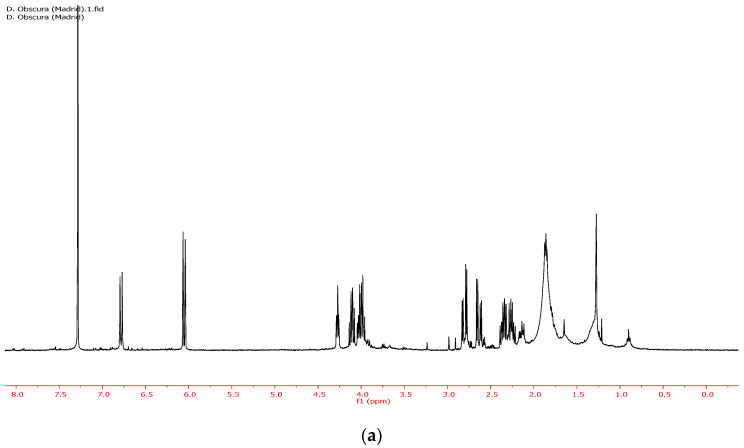

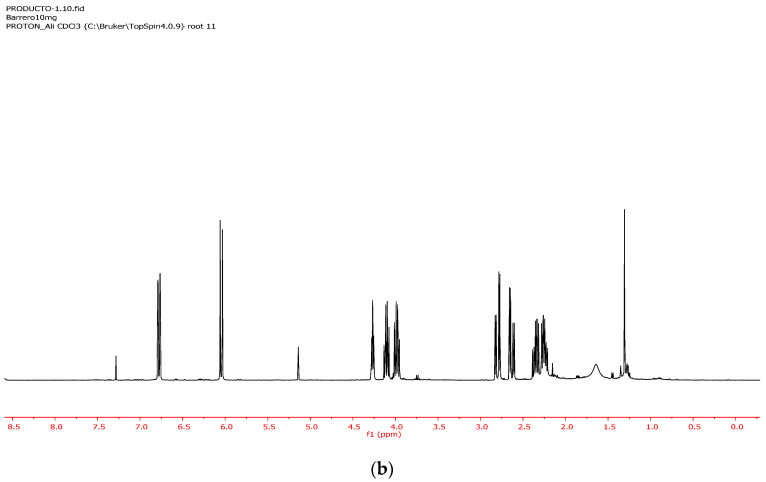
(**a**) ^1^H NMR of the CDCl_3_ soluble fraction of the ethanolic extract of *D. obscura*. (**b**) NMR of pure rengyolone.

Furthermore, the CHCl_3_ soluble fraction of the ethanolic extract (entry 5, Table 1) allowed for very efficient separation of rengyolone (**1**) from the sugar fraction. In fact, the ^1^HNMR spectra of the CHCl_3_ soluble fraction of the ethanol extract (Figure 2a) and pure rengyolone (**1**) (Figure 2b) were almost identical. 

When this CHCl_3_ soluble fraction was subjected to column chromatography, rengyolone was obtained along with a minor proportion of the saturated derivative of rengyolone, cleroindicin C (**4**) [30]. 

Rengyolone (or halleridone) (**1**) was first isolated from the medicinal plants *Forsythia suspensa* [31] and *Halleria lucida* [32]. Additionally, a compound with the same spectroscopic data of rengyolone/halleridone was isolated from *Clerodendrum indicum* and was named as cleroindicin F [30]. The synthesis of **1** using tyrosol as starting material has been described [33,34,35].

In summary, pure rengyolone (**1**) can be obtained from *D. obscura* in approximately 90% yield purity from fresh plant using only two simple lab operations. Up to 640 mg of rengyolone were obtained from 80 g of dry plant (0.8%).

### 2.3. Selective Extraction of Cornoside Aglycon (***3***)

Compound **3** is the aglycon of the known compound cornoside (**2**), whose presence was also confirmed in the refluxing ethanol extract of *D. obscura* (entry 2, Table 1).

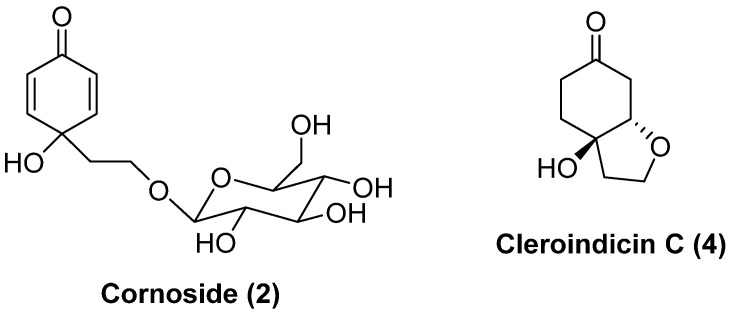



Cornoside (**2**) has been reported to be present in many species of Cornus [36] and Olea europea and Digitalis [12,37]. However, the occurrence of its aglycone, compound **3** has been always associated to rengyolone [12,38]. Possibly, due to this known instability of compound **3** [37,39], no ^13^C-NMR data of this compound and of its biological activity could be found in the literature. All these led us to study the selective extraction and isolation of compound **3** in order to study both its possible activity as biopesticide and stability. 

**Figure 3:**
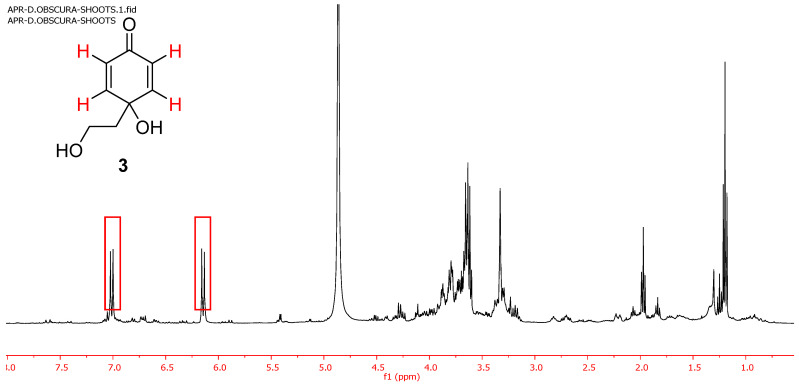
^1^H NMR spectrum of the ethanolic extract of *D. obscura* collected in March 2021.

The known predisposition of the cornoside aglycon (**3**) to evolve towards rengyolone led us to perform the extraction of freshly collected *D. obscura* to avoid the possible Michael addition converting **3** into **1** due to the plant drying process. The analysis of the ethanol extract of fresh plant samples collected in March 2021 (entry 6, Table 1) showed the presence of compound **3** as the only phenylethanoid component (Figure 3). Only one column chromatography allowed to obtain pure **3** (0.4% from the fresh plant).

With regard to the occurrence of compound **3**, Jensen et al. [38] reported that the presence of this substance is probably due to the action of glycosidases on cornoside (**2**). To verify this hypothesis, we conducted an extraction of *D. obscura* collected in March 2021 with refluxing ethanol-a process that should deactivate the corresponding glycosidases, to find that compound **3** was still the more abundant phenylethanoid although the presence of cornoside (**2**) was also observed. This presence is easily rationalized as a result of the increase of the capacity of extraction of polar components when the temperature of the solvent is raised. This observation suggests that *D. obscura* actually produces compound **3** naturally in the early stages of its growth, and that, eventually, this compound is glycosylated to generate cornoside (**2**). In this regard, the occurrence of cleroindicin C (**4**) in *D. obscura* suggest that, at least to some extent, the cyclization of cleroindicin C (**4**) to give rengyolone (**1**) could also take place in vivo.

Compound **3** was then acetylated. With this transformation, not only we could obtain the natural compound hallerone (**5**) [32], but also this could be a way of stabilizing compound **3** by precluding the intramolecular Michael addition leading to rengyolone (**1**). When the reaction was performed using acetic anhydride, mixtures containing the diacetylated compound (**6**) [40] were obtained. The acetylation of the tertiary alcohol was avoided by using acetyl chloride and collidine at −78 °C [41], with hallerone (**5**) being obtained in a 65% yield. 

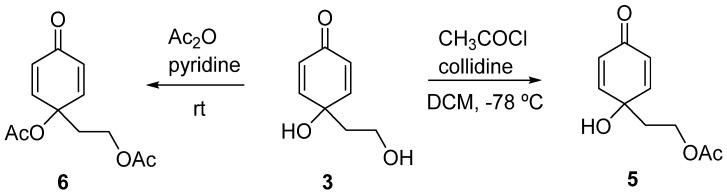



This compound was reported to possess antimicrobial and antifungal activity [42], showed moderate scavenging action on superoxide radicals, inhibited H_2_O_2_ induced reactive oxygen species production in HEK-293 cells [18], and its synthesis has been described by different authors [33,34,35].

### 2.4. Bioactivity of Phenylethanoids

Phenylethanoids **1**, **3**, **5,** and **6** were tested against *M. persicae* and *M. javanica* (Table 2 and Table 3). At the maximum dose tested (50 µg/cm^2^), compounds **1** (4 doses, 86–37 %SI), **3** (3 doses, 76–28 %SI) and **5** (4 doses, 78–35 %SI) were effective. The activity of compound **5**, with an efficient dose (EC_50_) of 12.5 µg/cm^2^ (7.0–22.0 95% CL) fell within the range of thymol (EC_50_ of 7.6, 4.1–8.7 95% CL) (Table 2), included as a positive control since this compound is an active ingredient of commercial biopesticides [5].

When these compounds were tested against *M. javanica*, hallerone **5** showed strong nematicidal effects (Table 3) with an LD_50_ of 0.034 mg/mL (Table 4), five times more effective than thymol, a reference compound with proven nematicidal activity (LD_50_ value of 0.14 mg/mL) [43].

This is the first report on the aphid antifeedant effects of **1**, **3,** and **5**. The aglycon of **3**, cornoside (**2**), had growth regulation effects on *Rhodnius nasutus* [45] and ethyljacaranone, structurally related to **5**, proved to be a potent antifeedant against S. littoralis and *M. persicae* while a jacaranone diol derivative only showed significant antifeedant effects on *M. persicae*, [46] supporting the aphid antifeedant effects of phelylethanoids. Additionally, the nematicidal activity of hallerone (**5**) is described here for the first time. It is interesting to note the structural specificity of this nematicidal effect. 

## 3. Conclusions

Cardenolide-free extracts from *Digitalis obscura* showed significant antifeedant effects against the aphid *Myzus persicae*, and this activity correlated with their phenylethanoid content.

Pure rengyolone (**1**) can be obtained from *D. obscura* in approximately 90% purity from fresh plant from the CHCl_3_ soluble fraction of the ethanolic extract (8% yield). The ethanol extraction of freshly collected *D. obscura* showed the presence of compound **3** as the only phenylethanoid. 

This is the first report on the aphid antifeedant effects of **1**, **3**, and **5**. Additionally, the nematicidal activity of hallerone (**5**) is described here for the first time.

The efficient and selective extraction of these substances, which avoids laborious and costly separations, along with their antifeedant and selective nematicidal effects points out their potential as biopesticide models.

## 4. Experimental Section

### 4.1. Plant Material

Specimens of *Digitalis obscura* L. were collected in Prado Negro (37.0850844, −3.3798640, Granada, Spain) in July 2020 and March 2021. Aerial plant parts (dried for 15 days or fresh) were obtained by maceration in different solvents (for more details and conditions, see Table 1). Extracts from powdered aerial parts were also obtained using a Soxhlet extractor for 12 h with a 7:3 mixture of EtOH:H_2_O. 

### 4.2. Selective Extraction of Rengyolone

Aerial parts of *D. obscura* (80 g) collected in July 2020 were immersed in EtOH and heated at 40 °C for 2 h. After evaporation of the solvent, 1.2 g of extract was obtained. The CHCl_3_ soluble fraction of this extract (870 mg) was flash chromatographed (H:MTBE 2:1) to give 640 mg of rengyolone (**1**) and 4 mg of compound **4**.

*Rengyolone* (**1**): ^1^H NMR (400 MHz, CDCl_3_) d = 6.78 (dd, *J* = 10.2, 1.5 Hz, 1H), 6.05 (d, *J* = 10.2 Hz, 1H), 4.27 (dd, *J* = 8.9, 1.5 Hz, 1H), 4.10 (td, *J* = 8.5, 6.5 Hz, 1H), 3.98 (td, *J* = 8.5, 6.3 Hz, 1H), 2.80 (dd, *J* = 16.9, 4.8 Hz, 1H), 2.63 (dd, *J* = 16.9, 5.9 Hz, 1H), 2.35 (ddd, *J* = 13.0, 8.4, 6.4 Hz, 1H), 2.25 (ddd, *J* = 13.0, 8.2, 6.5 Hz, 1H) (Figure S1a). ^13^C NMR (126 MHz, CDCl_3_) δ = 196.49 (C), 147.54 (CH), 128.82 (CH), 82.27 (CH), 75.80 (C), 66.26 (CH_2_), 40.24 (CH_2_), 39.61 (CH_2_). (Figure S1b).

*Cleroindicin C* (**4**): ^1^H NMR (400 MHz, CDCl_3_) δ = 4.03−3.96 (m, 2H), 3.91 (td, *J* = 8.9, 7.4 Hz, 1H), 2.75 (dd, *J* = 16.1, 4.8 Hz, 1H), 2.59 (ddd, *J* = 16.2, 4.7, 0.8 Hz, 1H), 2.50 (ddd, *J* = 17.4, 8.1, 5.1 Hz, 1H), 2.30 (dddd, *J* = 17.4, 8.6, 4.7, 0.9 Hz, 1H), 2.21–2.06 (m, 4H) (Figure S1f). ^13^C NMR (126 MHz, CDCl_3_) δ = 209.29 (C), 83.54 (CH), 65.95 (CH), 42.38 (CH_2_), 40.67 (CH_2_), 35.00 (CH_2_), 35.42 (CH_2_) (Figure S1g).

### 4.3. Selective Extraction of Compound ***3***

A total of 60 g of the fresh aerial parts of *D. obscura* collected in March 2021 were immersed in EtOH heated at 40 °C for 1 h. After evaporation of the solvent, 450 mg of extract was obtained. This extract was flash chromatographed (MTBE:EtOAc 2:1) to give 220 mg of compound **3**.

*Compound***3**. ^1^H NMR (400 MHz, MeOD) d = 7.01 (d, *J* = 10.2 Hz, 1H), 6.14 (d, *J* = 10.1 Hz, 1H), 3.66 (t, *J* = 6.7 Hz, 2H), 1.97 (t, *J* = 6.7 Hz, 2H) (Figure S1d). ^13^C NMR (126 MHz, MeOD) δ = 196.39 (C), 153.03 (2 × CH), 126.48 (2 × CH), 67.96 (C), 56.85 (CH_2_), 42.36 (CH_2_) (Figure S1e).

For acetylation of compound **3** with acetyl chloride, 0.07 ml of collidine (0.56 mmol) and 0.022 mL of acetyl chloride (0.28 mmol) were added to a solution of compound **3** (40 mg, 0.26 mmol) in 3 mL DCM cooled at −78 °C. The mixture was stirred for 2 h at this temperature. It was then poured into 2N HCl and extracted with MTBE (20 mL × 3). The combined organic layers were dried over anhydrous sodium sulfate and concentrated in vacuo. The resultant crude was purified by flash chromatography (H: MTBE 1:1) to give hallerone (**5**) (33 mg, 65%).

*Hallerone* (**5**). ^1^H NMR (400 MHz, CDCl_3_) δ = 6.80 (bd, *J* = 10.2 Hz, 1H), 6.12 (bd, *J* = 10.2 Hz, 1H), 4.10 (t, *J* = 6.5 Hz, 2H), 2.04 (t, *J* = 6.6 Hz, 2H), 1.96 (s, 3H) (Figure S1i). ^13^C NMR (126 MHz, CDCl_3_) δ = 185.06 (C), 170.70 (C), 147.47 (2 × CH), 128.99 (2 × CH), 75.20 (C), 59.11 (CH_2_), 38.47 (CH_2_), 21.22 (CH_3_), 20.86 (CH_3_) (Figure S1j).

Acetylation of compound **3** with Ac_2_O. To a solution of compound **3** (20 mg, 0.13 mmol) in 1 mL pyridine cooled at 0 °C, 0.01 mL of Ac_2_O and a crystal of DMAP were added. The mixture was stirred for 30 min at this temperature and then 2 h at rt. The reaction mixture was then poured into ice and extracted with MTBE (20 mL × 3). The combined organic layers were washed with 2N HCl (10 mL × 3), saturated NaHCO_3_ (20 mL × 3), and brine (20 mL × 3). Then, it was dried over anhydrous sodium sulfate and concentrated in vacuo. The resultant crude was purified by flash chromatography (H: MTBE 4:1) to give compound **6** (16 mg, 52%).

*Compound***6**. ^1^H NMR (400 MHz, CDCl3) δ = 6.88 (d, *J* = 10.2 Hz, 1H), 6.29 (d, *J* = 10.2 Hz, 1H), 4.18 (t, *J* = 6.6 Hz, 2H), 2.17 (t, *J* = 6.6 Hz, 2H), 2.07 (s, 3H); 2.03 (s, 3H) (Figure S1k). ^13^C NMR (126 MHz, CDCl_3_) δ = 184.81 (C), 170.60 (C), 169.19 (C), 150.17 (2 × CH), 128.27 (2 × CH), 68.40 (C), 59.60 (CH_2_), 38.72 (CH_2_), 20.87 (CH_3_) (Figure S1l).

### 4.4. Antifeedant Activity

*S. littoralis*, *M. persicae* and *R. padi* colonies are maintained at ICA-CSIC, reared on artificial diet, bell pepper (*Capsicum annuum*) and barley (*Hordeum vulgare*) plants, respectively, and kept at 22 ± 1 °C and >70% RH, with a photoperiod of 16:8 h (L:D) in a custom-made walk-in growth chamber.

The bioassays were conducted as described [43]. The upper surface of *C. annuum* and *H. vulgare* leaf disks or fragments (1.0 cm^2^) were treated with 10 µL of the test substance. The extracts and products were tested at an initial dose of 10 or 5 µg/µL (100 or 50 µg/cm^2^) respectively. A total of 5 to 7 Petri dishes or 20 ventilated plastic boxes (2 × 2 cm) with 2 sixth-instar *S. littoralis* larvae (>24 h after molting) or 10 apterous aphid adults (24–48 h old) each were allowed to feed in a growth chamber (until 75% larval consumption of control disks or 24 h for aphids, environmental conditions as above). Each experiment was repeated 2–3 times. Feeding inhibition or aphid settling was calculated by measuring the disk surface consumption (digitalized with https://imagej.nih.gov/ij/ (accessed on 3 March 2021) [47]) or by counting the number of aphids on each leaf fragment. Feeding/Settling inhibition (%FI or %SI) was calculated as % FI/SI = [1 − (T/C) × 100], where T and C represent feeding/settling on treated and control leaf disks, respectively. The antifeedant effects (% FI/SI) were analyzed for significance by the nonparametric Wilcoxon paired signed-rank test comparing the consumption/settling between the treatment and control leaf disks. Extracts and compounds with an SI > 70% were further tested in a dose-response experiment (1:2 serial dilutions to cover a range of activities between 100 and <50% feeding inhibition with a minimum of 3 doses) to calculate their effective dose EC_50_ (dose to give a 50% settling reduction) from linear regression analysis (% FI/SI on Log-dose, STATGRAPHICS Centurion XVI, version 16.1.02).

### 4.5. Nematicidal Bioassay

The *M. javanica* population was maintained on *Lycopersicon esculentum* plants (var. Marmande) in pot cultures at 25 ± 1 °C, 70% relative humidity. Egg masses of *M. javanica* were hand-picked from infected tomato roots. Second-stage juveniles (J2) were obtained from hatched eggs by incubating egg masses in a water suspension at 25 °C for 24 h. Bioassays were performed in 96-well plates (BD Falcon, San Jose, CA, USA) as described by Andrés et al. [48]. Extracts and compounds were dissolved in water with 5% of DMSO-Tween solution (0.5% Tween 20 in DMSO), 5 µL of this solution was added to 95 µL of water containing 90–100 nematodes to obtain an initial concentration of 1 mg/mL per well. Treatments were replicated 4 times. As a control, 4 wells were filled with the water/DMSO/Tween 20 in the same volume as the test solutions. The plates were covered to prevent evaporation and were maintained in the dark at 25 °C. After 72 h, the dead J2 were counted under a binocular microscope. The nematicidal activity data were presented as percent dead J2s corrected according to Schneider-Orelli’s formula [44]. Five serial concentrations of the active compound were tested to obtain an effective lethal dose (LD50) by Probit analysis (STATGRAPHICS Centurion XVI, version 16.1.02).

## Figures and Tables

**Table 1:** Qualitative content in different extracts of *D. obscura* ^a^.

Entry	Method	Fats	Phenyletanoids	Phenyletanoids (Glycosylated)	Cardenolides	Free Sugars
1	70% EtOH (reflux) ^b^	tr ^e^	+	+ +	+	+ +
2	EtOH (reflux)	tr	+	+ +	+	+ +
3	MTBE (rt ^c^)	+ +	+	−	−	−
4	EtOAc (rt)	+ +	+	−	−	−
5	EtOH (40 °C)	tr	+ +	−	−	+
6	EtOH (40 °C) ^d^	tr	+ +	−	−	+

^a^ Dried during 15 days. ^b^ Powdered aerial parts. ^c^ Room temperature. ^d^ Fresh plants (extracted the day after the collection). ^e^ Traces).

**Table 2:** Antifeedant effects of compounds **1**, **3**, **5** and **6** against *Myzus persicae*.

Compound	% SI ^a^	EC_50_^b^ (µg/cm^2^)
**1**	86.31 ± 5.74 *	17.6 (13.2–23.4)
**3**	75.77 ± 6.13 *	25.9 (18.5–36.1)
**5**	76.81 ± 5.51 *	12.5 (7.0–22.0)
**6**	53.78 ± 8.58	
**Thymol**	81.8 ± 7.7 *	7.6 (4.1–8.7)

^a^ Percent setting (SI) inhibition at a dose of 100 μg/cm^2^. Values are means of twenty replicates. Values with asterisk (*) are significantly different according to Wilcoxon paired rank test (*p* < 0.05). ^b^ Effective dose EC_50_ (95% lower-upper confidence limits) needed to produce 50% feeding/setting inhibition.

**Table 3:** Nematicidal activity of compounds **1**,**3**, **5**, and **6** against *M. javanica* juveniles.

Compound	% Mortality ^a^
**1**	34.17 ± 10.81
**3**	2.47 ± 1.56
**5**	92.87 ± 1.26
**6**	20.34 ± 2.52

^a^ Percent mortality at a dose of 0.5 mg/mL. Values (%) are means of four replicates (corrected according to Schneider–Orelli’s formula [44].

**Table 4:** Comparative nematicidal effects of active compound **5** and thymol against *M. javanica* juveniles.

Compound	Dose (µg/mg)	J2 Mortality (%) ^a^	LD_50_^b^
**5**	1.0	92.87 ± 1.23	0.034 (0.017–0.091)
	0.5	82.13 ± 4.1	
	0.25	75.83 ± 1.39	
	0.12	46.82 ± 5.11	
	0.06	40.93 ± 2.59	
**Thymol**	1.0	100	0.14 (0.131–0.143)
	0.50	100	
	0.25	98 ± 0.44	
	0.12	29.14 ± 2.92	
	0.06	15.40 ± 2.03	

^a^ Percent mortality at a dose of 0.5 mg/mL. Values (%) are means of four replicates (corrected according to Schneider-Orelli’s formula [44]. ^b^ Lethal dose in mg/mL (upper-lower 95% confidence limits) calculated to give 50% (LD_50_) mortality by Probit Analysis.

## Supplementary Materials

The following are available online at https://www.mdpi.com/article/10.3390/plants10050959/s1, Figure S1. copies of NMR spectra of compounds **1**, **3**, **4**, **5**, and **6**.
