# Supplementary material for: Selective Extraction of Bioactive Phenylethanoids from Digitalis obscura
Source: Plants (Basel). 2021 May 12;10(5):959. doi: 10.3390/plants10050959 (PMC8150932; doi:10.3390/plants10050959)
Supplement: Supplementary file 1 [file plants-10-00959-s001.zip › plants-1200743-supplementary.pdf]

Selective Extraction of bioactive phenylethanoids from *Digitalis obscura*

José Francisco Quílez del Moral [1,*], Álvaro Pérez [1], María José Segura Navarro [1], Alberto Galisteo [1], Azucena Gonzalez-Coloma [2], María Fe Andrés [2] and Alejandro F. Barrero [1,*]

[1] Department of Organic Chemistry, Institute of Biotechnology, University of Granada, 18071 Granada, Spain; alvaroapr@ugr.es (A.P.) albertogapre@ugr.es (A.G.); mariajoseseguranavarro@gmail.com (M.J.S.N.).

[2] Institute of Agricultural Sciences, CSIC, 28006, Madrid, Spain; azu@ica.csic.es (A.G.C.); mafay@ica.csic.es (M.F.A).

* Correspondence: jfquilez@ugr.es; afbarre@ugr.es; Tel.: +34-958243185

List of contents

Figure S1. Copies of NMR spectra 2-8

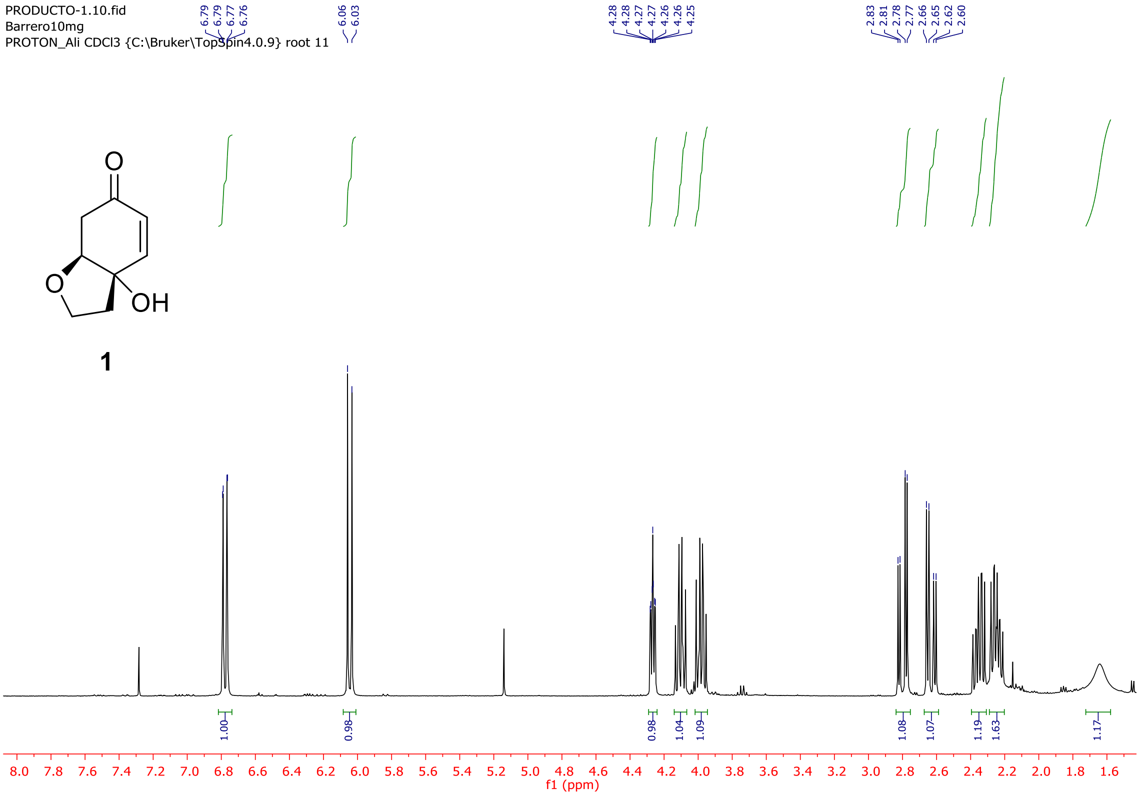

Figure S1a. ^{1}H NMR spectrum of compound **1**

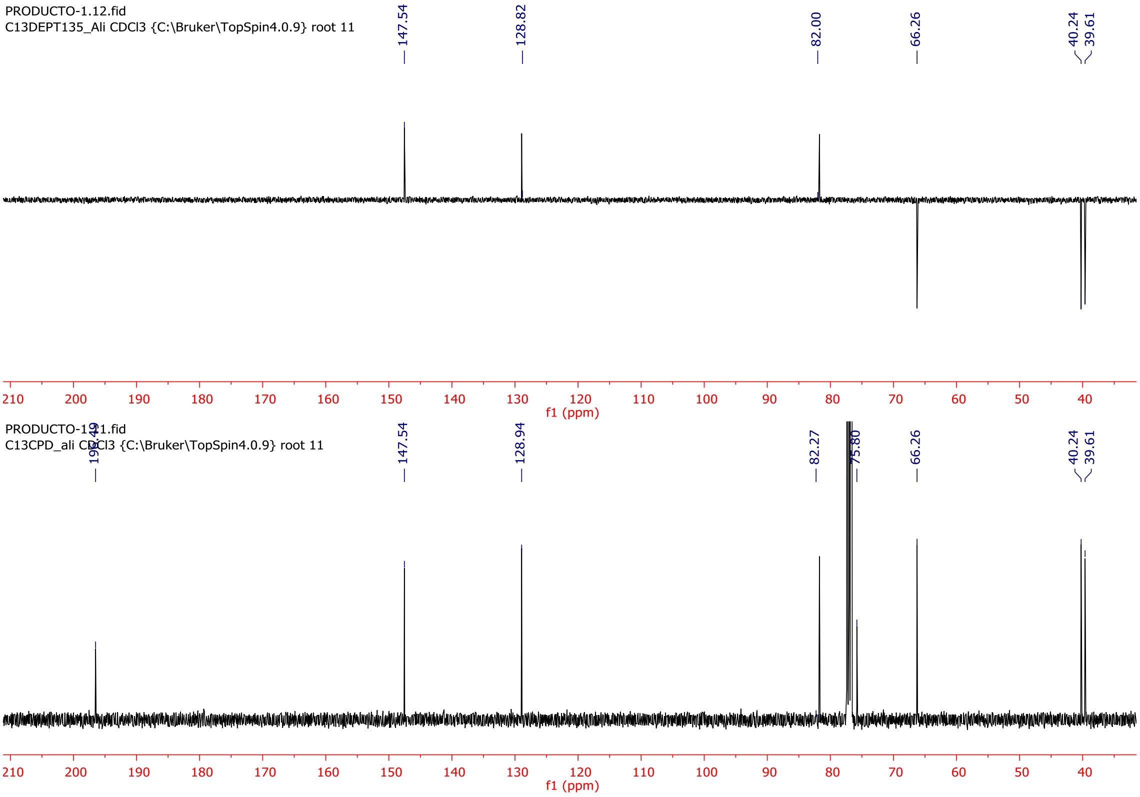

Figure S1b. ^{13}C NMR spectrum of compound **1**

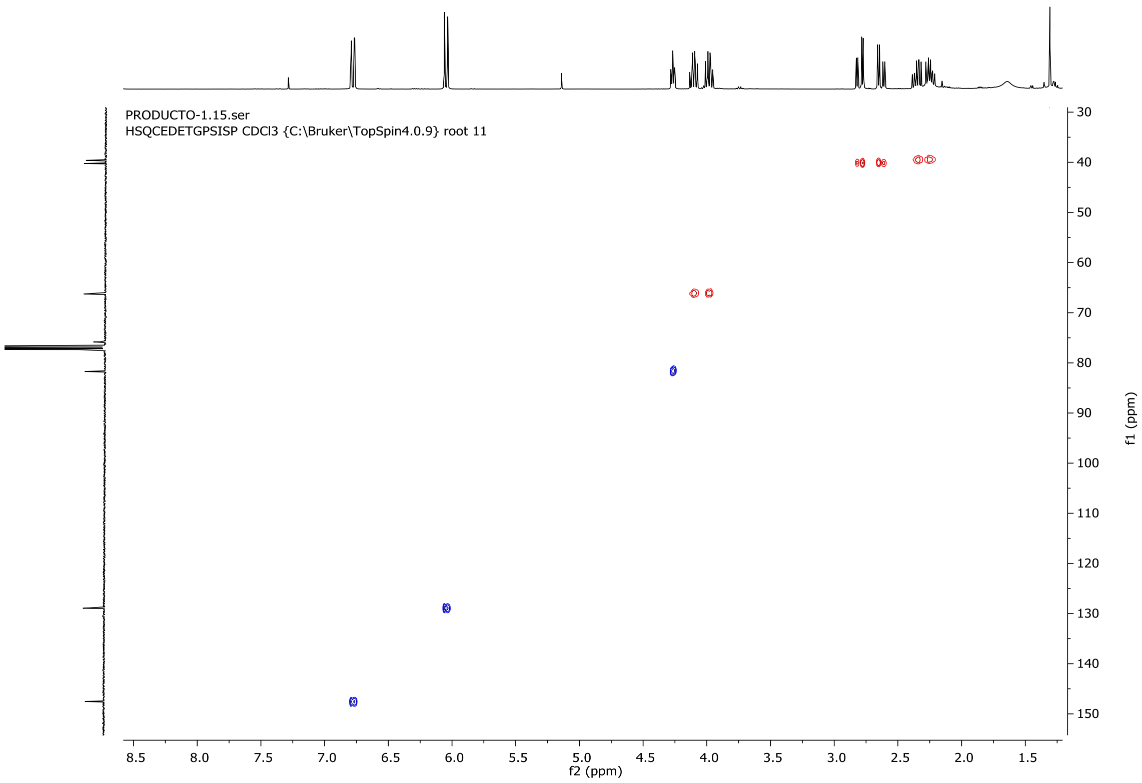

Figure S1c. HSQC of compound **1**

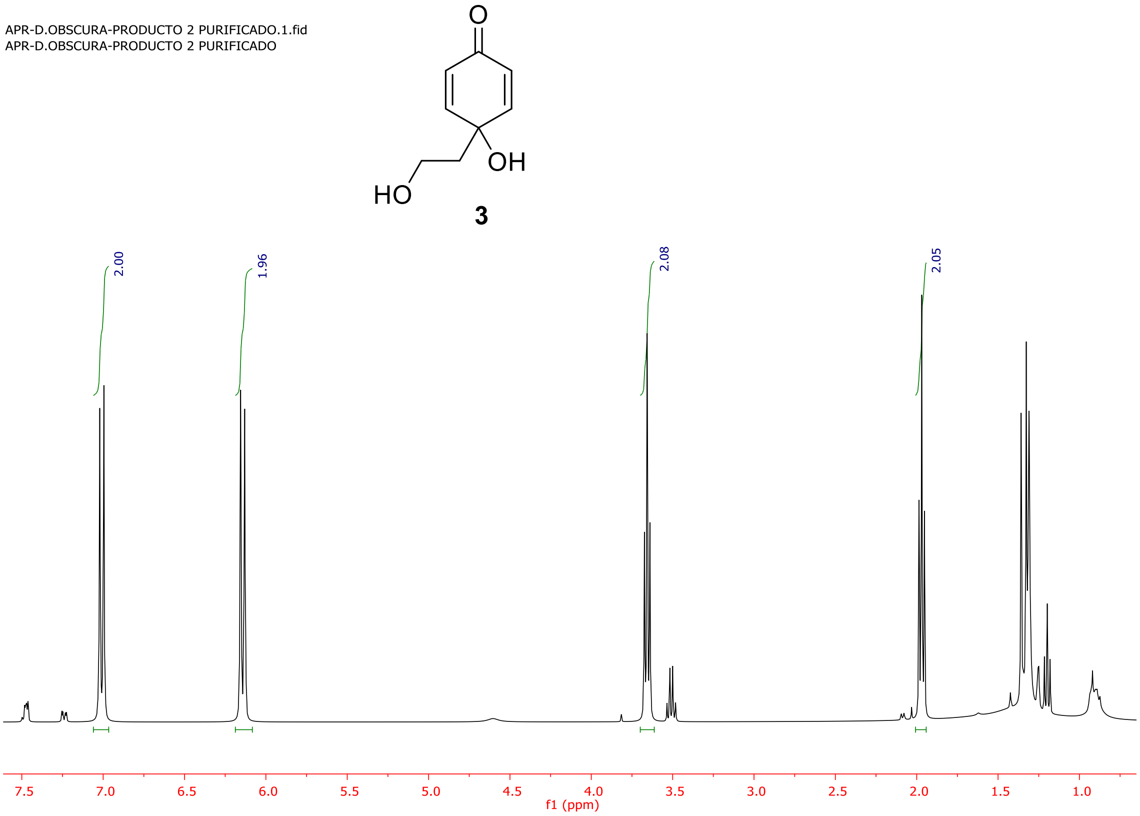

Figure S1d. ^{1}H NMR spectrum of compound **3**

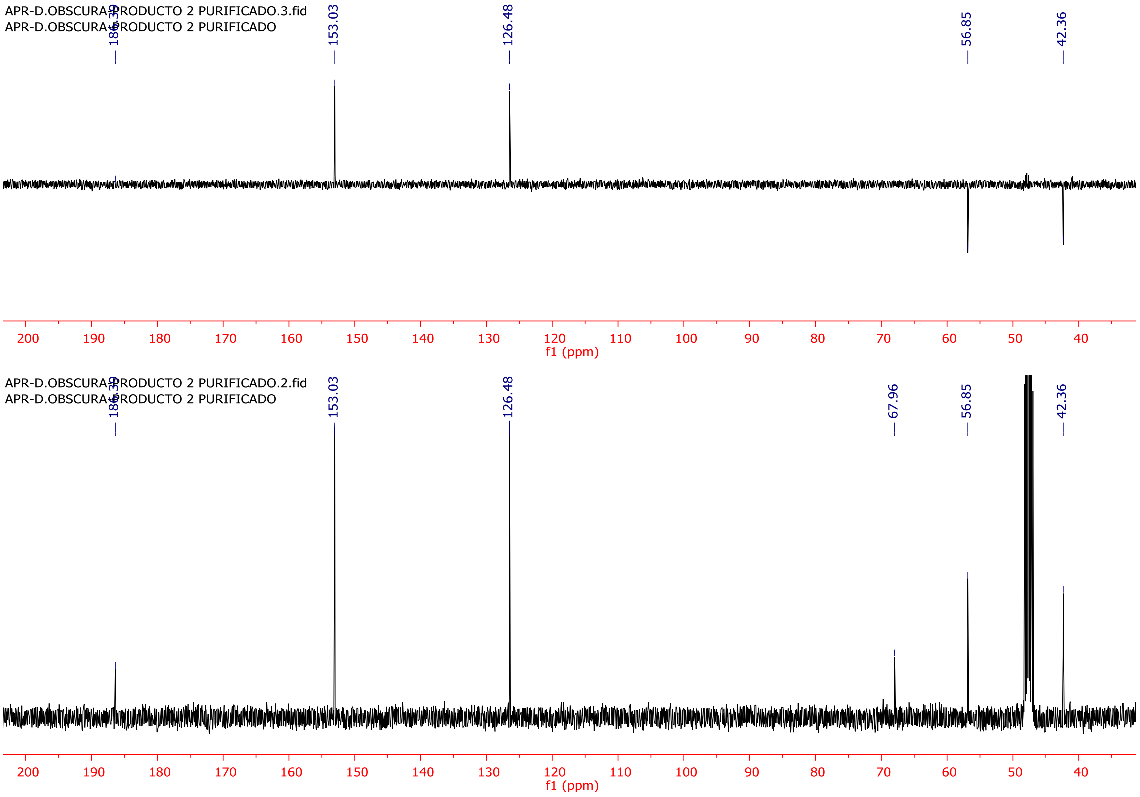

Figure S1e. ^{13}C NMR spectrum of compound **3**

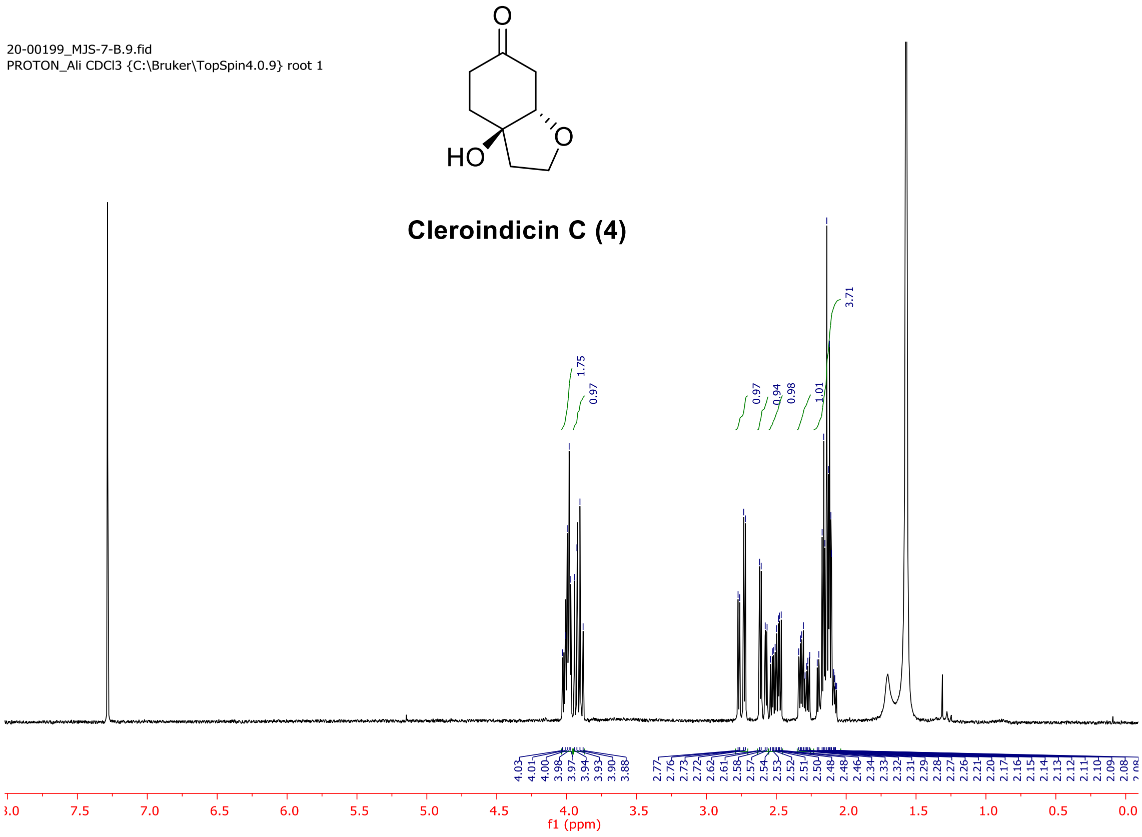

Figure S1f. ^{1}H NMR spectrum of compound **4**

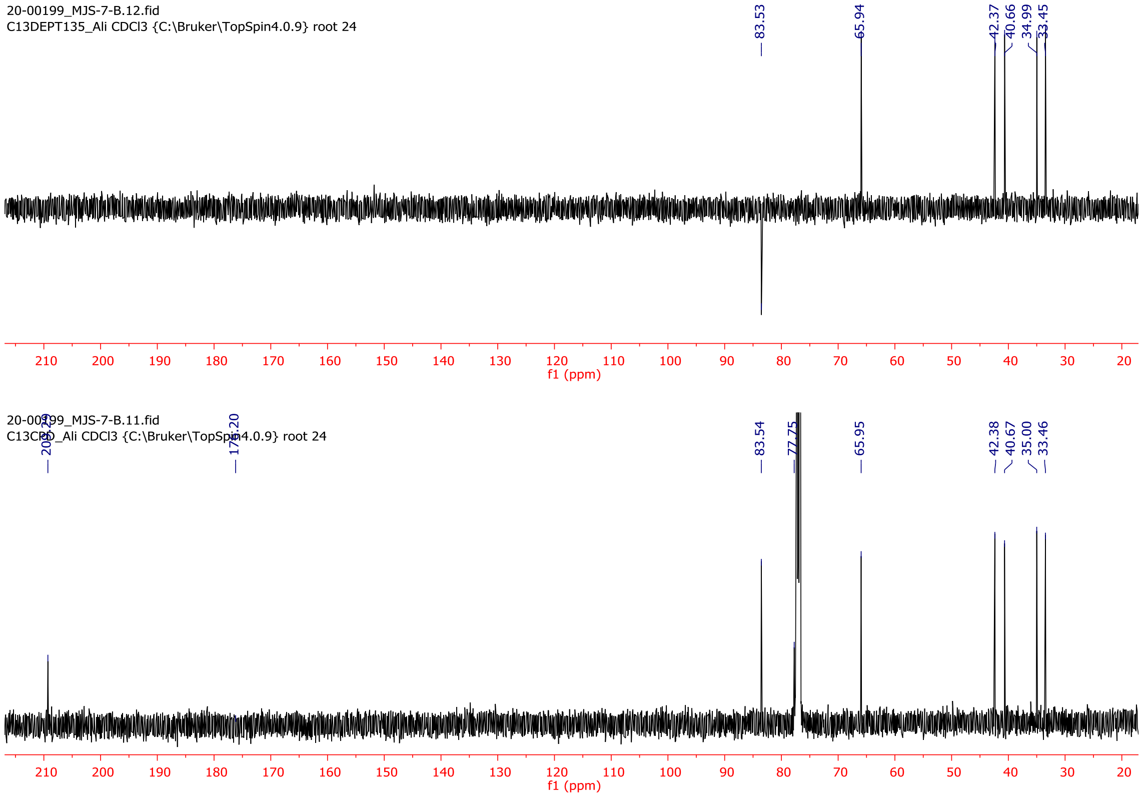

Figure S1g. ^{13}C NMR spectrum of compound **4**

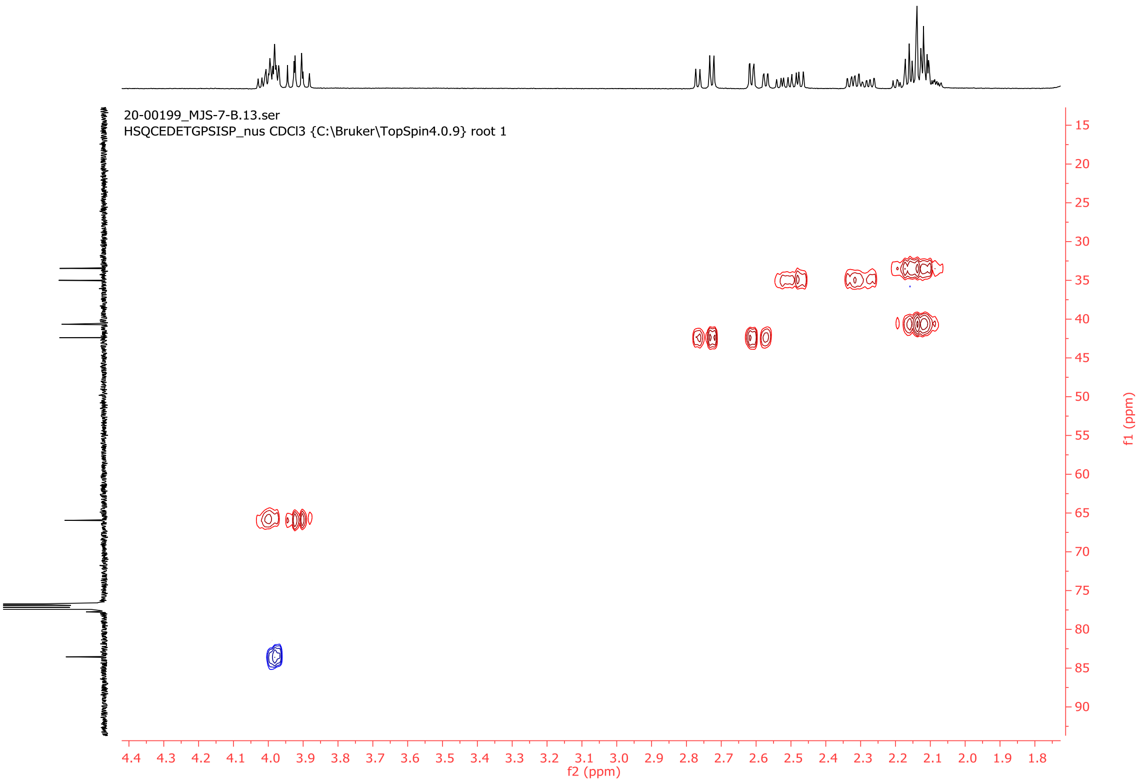

Figure S1h. HSQC of compound **4**

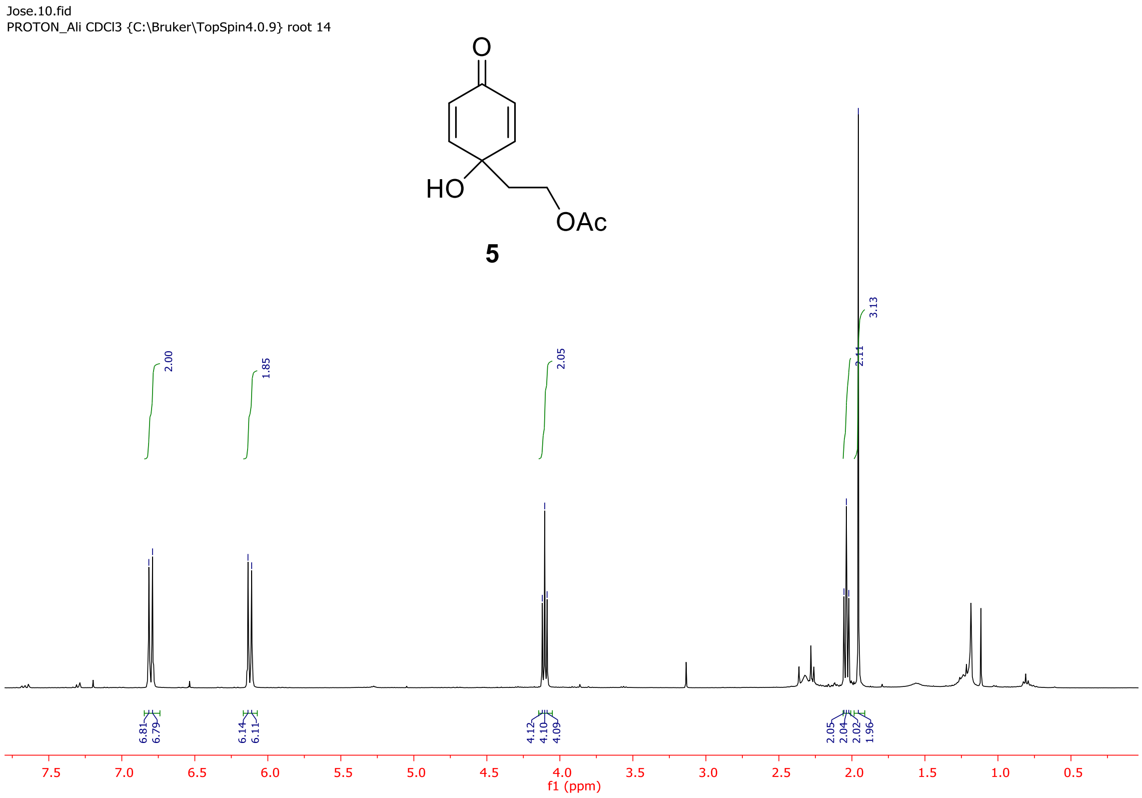

Figure S1i. ^{1}H NMR spectrum of compound **5**

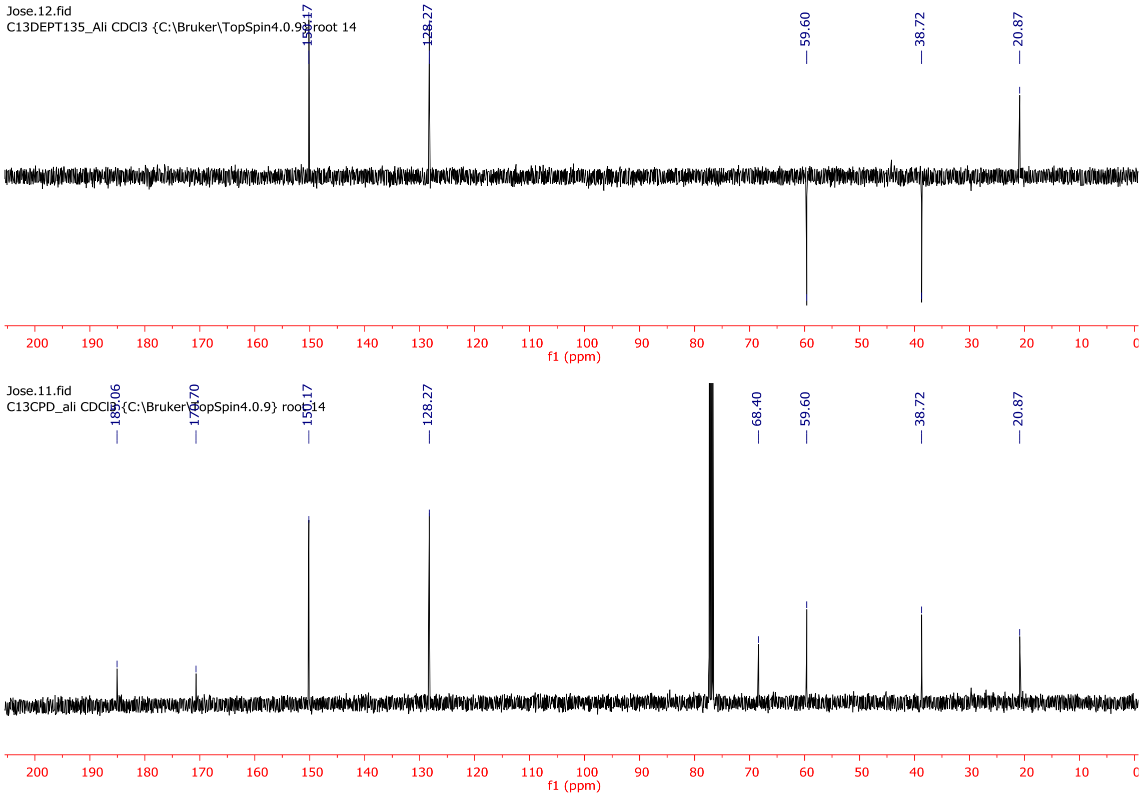

Figure S1j. ^{13}C NMR spectrum of compound **5**

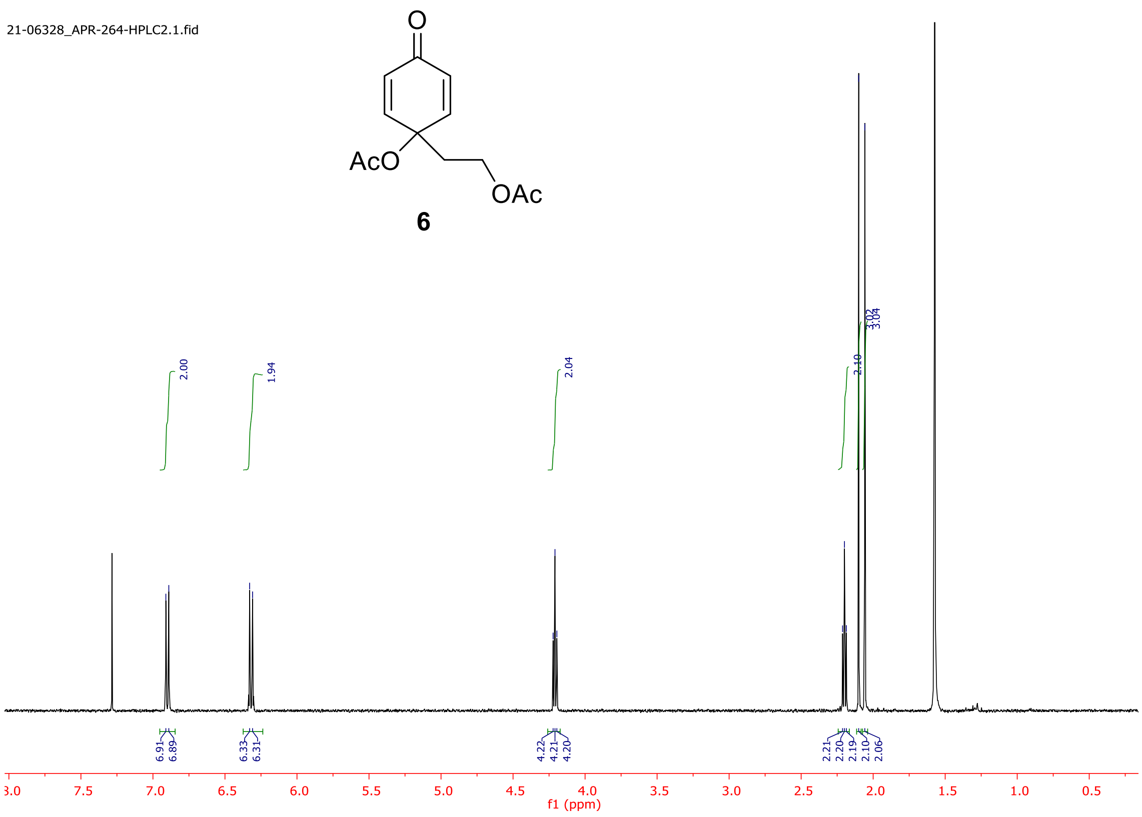

Figure S1k. ^{1}H NMR spectrum of compound **6**

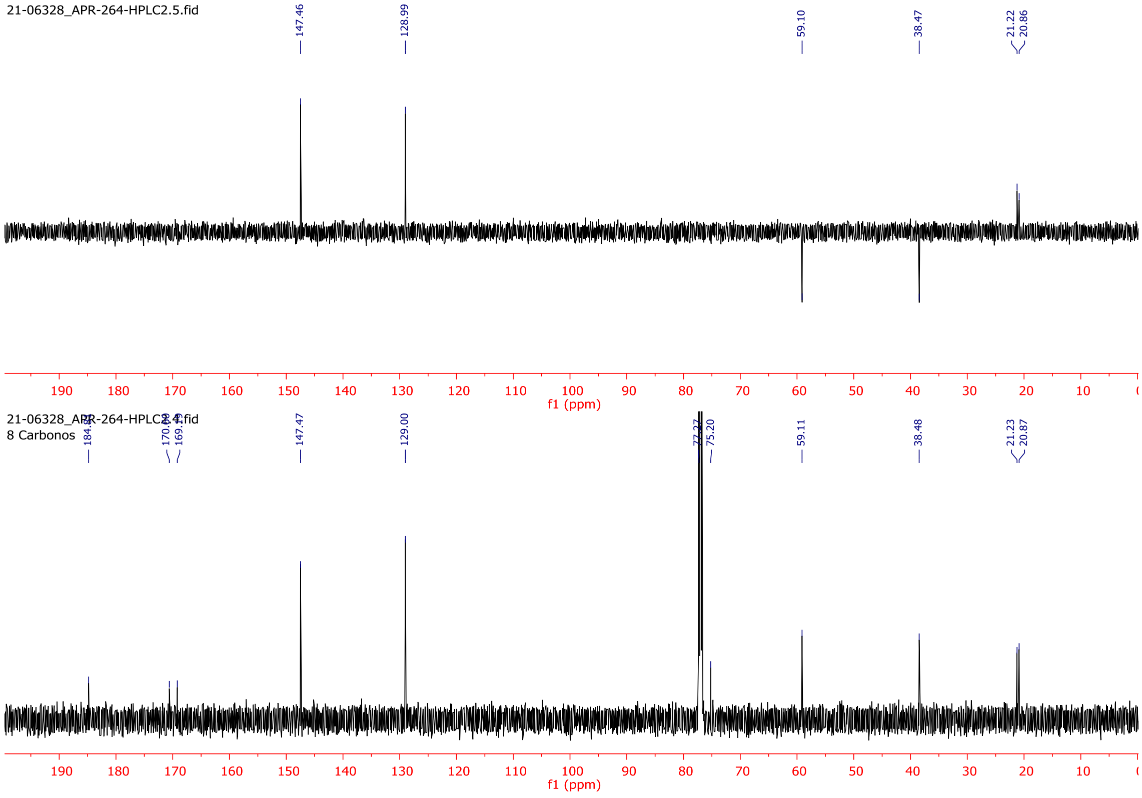

Figure S1l. ^{13}C NMR spectrum of compound **6**
